# Study on the types of abuse in young couples as a function of sex

**DOI:** 10.3389/fpsyg.2023.1166834

**Published:** 2023-07-21

**Authors:** Gracia González-Gijón, Francisco Javier Jiménez-Rios, Nazaret Martínez-Heredia, Andrés Soriano Díaz

**Affiliations:** Department of Pedagogy, Faculty of Education, University of Granada, Granada, Spain

**Keywords:** violence, young couples, university students, sex, quantitative

## Abstract

This study analyses the types of violence that can occur in intimate partner relationships among young people and their self-perception of abuse. For this purpose, we have used a survey-type methodology, with a quantitative approach. Participants were selected by means of non-probabilistic convenience and consisted of students enrolled in different degree and postgraduate courses in the Faculty of Education Sciences of the University of Granada (Spain). The sample consisted of 323 students, with a mean age of 23.8 years (SD = 5.2). Statistical and inferential tests were carried out with the data obtained using the SPSS V26 data analysis programme. The results show that the type of maltreatment most suffered, at some time by the sample participants, is emotional maltreatment, physical maltreatment, and psychological maltreatment. By comparing the means obtained, we can conclude that sex did not influence the violence suffered by young couples, which gives it a bidirectional character.

## 1. Introduction

Dating violence can be defined as the set of verbal, psychological, physical, and/or sexual behaviours that take place in young couples, whether they are more or less long-lasting or short-term. This includes relationships between non-marital partners, including first dates, as well as heterosexual and homosexual relationships (Hamby and Turner, 2013; Pazos Gómez et al., 2014; Rubio et al., 2017; Pérez-Marco et al., 2020).

Violence in intimate partner relationships has two key characteristics. First, in specific terms, it occurs more frequently, and second, it is closely related to a relationship characterised by conflict and power; therefore, the correlation between victimisation and violence is very high (Zweig et al., 2014; Del Moral et al., 2020).

Violence in relationships is also quite widespread and a very important public health problem (Navarro-Pérez et al., 2020). Several research studies (Romera Félix et al., 2017; Carrascosa et al., 2018; Martín and De la Villa Moral, 2019; Dodaj et al., 2020; Taquette et al., 2020) have shown how this type of violence affects the health and lifestyle of the young population.

In the short term, violence has a far-reaching psycho-emotional impact, with victims tending to report lower rates of self-esteem, more pain, anxiety, guilt, and communication problems, and poorer problem-solving skills. Similarly, perpetration of this type of violence, in the long term, leads to mental health problems, including depressed mood, suicidal ideation, and eating disorders, resulting in psychopathological problems, such as somatisation, psychosis, paranoid ideation, and post-traumatic stress disorder (Franco et al., 2012; Dillon et al., 2013: Díaz-Aguado and Martínez, 2016; Nascimento et al., 2018; Caba et al., 2019).

In the World Health Organization [WHO], 2013 established mental health as a priority public health problem and set out guidelines and plans to address it, including action on issues related to violence in relationships.

Similarly, in, the World Health Organization [WHO] (2017) recognised that this type of violence is a health problem in different parts of the world, and for this reason, policies and action programmes have been implemented (Díaz et al., 2020). This type of violence must continue to be addressed in prevention programming, as it most often occurs in a mixed-sex format, as well as improving the effectiveness of programming efforts aimed at reducing it, based on a critical understanding of gender differences and similarities in its perpetration (Dardis et al., 2015). All of this implies working at all levels of education; however, when talking about educational levels or stages, the university level is relegated and forgotten (Puigvert, 2008).

Numerous research studies (Valls et al., 2016; Boira et al., 2017; García-Carpintero et al., 2018; Kaufman et al., 2019; Tasayco et al., 2019; Osuna-Rodríguez et al., 2020) highlight the importance of researching and intervening in violence in young couples’ relationships at the university stage, given that training in the recognition of violent behaviours is very important for reducing it. These studies show that, during this stage, young people are at a high risk of experiencing this type of violence. For this reason, there is a need for more longitudinal research covering multiple experiences of violence victimisation among university students.

Similarly, a greater critical understanding of gender differences and the similarities in the perpetration of violence in young couples is needed to refine and improve knowledge about this issue (Valls et al., 2016; Wincentak et al., 2017; Sianko et al., 2019). Therefore, this study aims to analyse the violence that occurs in young couples and students. The main objective of this study is to analyse the prevalence of the types of violence that occur in young university students at the University of Granada, Spain, and examine the differences in the types of violence suffered according to sex.

## 2. Materials and methods

### 2.1. Design

The study presented here is descriptive and based on a survey-type methodology, with a quantitative approach.

### 2.2. Participants

The participants in this study were selected through non-probability or convenience sampling (Otzen and Manterola, 2017), in which all the students who were taught by the researchers in this study during the 2019–2020 academic year were invited to participate.

The study sample consisted of 323 students from the Faculty of Education Sciences at the University of Granada, Spain. Specifically, the young people were students enrolled in the master’s degree in research, social development, and socio-educational intervention (17.9%) and the degrees in social education (41.5%), paedagogy (30.6%), primary education (6.6%), and early childhood education (3.4%). The sample consisted of 90.1% women and 9.9% men, aged between 17 and 29 years, with a mean age of 23.8 (SD = 5.2). Of these, 57.9% do not live or have lived with the partner, and 42.1% live or have lived, with the partner referred to when answering the questions. Regarding the self-perception of abuse variable, 2.5% (*n* = 8) did have self-perception of abuse and 97.5% (*n* = 315) did not. Similarly, 73.1% (*n* = 236) did not perceive abuse in the past and 26.9% (*n* = 87) did.

### 2.3. Measuring instruments

The instrument used for data collection was the questionnaire on violence in young intimate relationships (VIREPA), by González-Gijón and Soriano (2021). This instrument consists of two parts that analyse, on the one hand, the socio-demographic variables divided into demographic factors, data on the partner and self-perception of abuse and, on the other hand, the remaining variables (20 in total), which measure the types of abuse that can occur in relationships and are grouped into five dimensions.

These dimensions are Emotional Abuse, Physical and Psychological Abuse, Personal Devaluation, Social and Economic Control, and Sexual Abuse. The dimensions according to González-Gijón and Soriano (2021) are defined as follows:

#### 2.3.1. Emotional mistreatment (EM)

This dimension consists of four items, which allow us to evaluate the psychological abandonment that involves the absence of attention to the affective needs and moods of the person and the form of mistreatment that is exercised through contemptuous forms that try to convince the other member of the relationship that they have low individual and social value.

#### 2.3.2. Physical and psychological mistreatment (PPM)

This dimension consists of five items that allow the detection of physical mistreatment, which is defined as any action or omission, not accidental, that causes physical harm to the person or places them at risk of suffering harm. Psychological mistreatment is defined as any behaviour that produces devaluation, suffering, or psychological harm, and the Münchausen syndrome, which occurs in situations in which fictitious symptoms and/or pathologies are fabricated or induced and are actively generated by the partner.

#### 2.3.3. Personal devaluation (PD)

This dimension consists of four items. This form of abuse attempts to devalue the person’s religious beliefs and ideological values while emphasising gender roles and stereotypes.

#### 2.3.4. Social and economic control (SEC)

This dimension consists of five items and identifies social control, which consists of surveillance, obstacles, and prohibitions that are put in place to hinder or prevent the interpersonal relationships of the partner, as well as economic control or abuse, which is understood as the use, without consent and in an abusive manner, of the objects of the other partner.

#### 2.3.5. Sexual abuse (SA)

This dimension consists of two items that identify the existence of abusive behaviours of a sexual nature, carried out from a position of power, without consent, and against the will of the partner, as well as the implementation of sexual behaviours that the other person regards as degrading and humiliating to their dignity (p. 7–8).

Cronbach’s Alpha statistic, with a value of α = 0.937, showed the scale had high reliability (Merino-Soto, 2016). The questionnaire is assessed through a Likert-type scale with five response options [1, never; 2, sometimes (1–2); 3, many times (3–5); 4, almost always (6 or more); and 5, always].

### 2.4. Procedure

For the collection of information, the Ethics Committee on Human Research (CEIH) of the University of Granada, Spain, was asked to evaluate the project. Once a favourable report was obtained, the instrument was prepared to be administered online using Google Forms software. This modality was chosen because of the circumstances of confinement in which we found ourselves due to the COVID-19 pandemic. This process was carried out during the 2019–2020 academic year.

### 2.5. Data analysis

Data analysis was performed with the SPSS v.26 statistical package (Stehlik-Barry and Babinec, 2017), using descriptive and inferential statistical techniques with parametric tests, given the normal distribution of the data previously found with the study of the normality of the sample through the Kolmogórov-Smirnov test, obtaining a value of *p* > 0.05, specifically 0.294. Student’s *t*-test was used, in which the grouping variable was sex.

## 3. Results

The percentage of agreement with certain items presented by pupils in each of the dimensions of the VIREPA questionnaire is shown below.

In relation to the factor of Emotional Mistreatment (Table 1), between 62 and 72% of the participants stated that they had never received this type of mistreatment. The item “shows indifference to your problems or needs” was the situation most experienced by the participants, with 22.9% having experienced it some of the time and 4.3% always. At the other extreme, the item “ridicules you or does not value you in front of other people” was the item with the lowest percentage (17% in the option “sometimes” and 1.2% “always”).

**TABLE 1:** Emotional abuse.

Items	Never		Ever (1–2)		Many times (3–5)		Almost always (6 or more)		Always	
	*f*	*%*	*f*	*%*	*f*	*%*	*f*	*%*	*f*	*%*
Is indifferent to your problems or needs	202	62.5	74	22.9	26	8.0	7	2.2	14	4.3
Does not value the work or effort you make	205	63.5	64	19.8	32	9.9	11	3.4	11	3.4
Does not take your opinion into account, does not consider your requests	209	64.7	68	21.1	19	5.9	14	4.3	13	4.0
Ridicules you or does not value you in front of other people	234	72.4	55	17.0	21	6.5	9	2.8	4	1.2

In relation to the factor of Physical and Psychological Abuse (Table 2), the percentages of participants who selected the option “never”, ranged between 67 and 92%, which were very high values and in contrast to the option “always,” which ranged between 0.3 and 2.8%. The item with the highest percentage was “sometimes their behaviour scared them,” which 21% of the participants never suffered (the second-most common type of abuse), followed by “insulting or threatening you” (14.2%).

**TABLE 2:** Physical and psychological abuse.

Items	Never		Ever (1–2)		Many times (3–5)		Almost always (6 or more)		Always	
	*f*	*%*	*f*	*%*	*f*	*%*	*f*	*%*	*f*	*%*
Sometimes their behaviour frightens you	218	67.5	68	21.1	23	7.1	8	2.5	6	1.9
Insults or threatens you	233	72.1	46	14.2	25	7.7	10	3.1	9	2.8
Tries to make you think you are sick	264	8.7	24	7.4	20	6.2	10	3.1	5	1.5
When he/she gets angry, he/she pushes you	279	86.4	29	9.0	10	3.1	4	1.2	1	0.3
Physically assaults you	298	92.3	20	6.2	4	1.2	1	0.3		

In the Personal Devaluation factor (Table 3), the percentages of participants who selected the option “never” ranged between 77 and 85%, which are also very high values. The option “always” had a maximum value of 2.5%. The item with the highest percentage was “ironises, ridicules your political ideology or religious beliefs,” which 14.2% of the participants experienced at some point, followed by “ridicules or insults you because you are a man or a woman” (10.5%).

**TABLE 3:** Personal devaluation.

Items	Never		Ever (1–2)		Many times (3–5)		Almost always (6 or more)		Always	
	*f*	*%*	*f*	*%*	*f*	*%*	*f*	*%*	*f*	*%*
Ironises, ridicules your political ideology or religious beliefs	251	77.7	46	14.2	17	5.3	4	1.2	5	1.5
Disrespects your political ideology or religious beliefs	263	81.4	27	8.4	17	5.3	8	2.5	8	2.5
Ridicules or insults you because you are a man or a woman	277	85.8	34	10.5	9	2.8	2	0.6	1	0.3
Forces you to perform tasks that he/she considers “your gender”	275	85.1	33	10.2	11	3.4	4	1.2		

As shown in Table 4, in relation to the Social and Economic Control factor, between 70 and 92% of the participants stated that they had never received this type of abuse. The item “controls your social networks and/or telephone” was the situation most experienced by the participants, with 16.7% having experienced it some of the time and 3.1% always. At the other extreme, the item “controls your money” was the one with the lowest percentage (5.3% for the option “sometimes” and 0.6% for “always”).

**TABLE 4:** Social and economic control.

Items	Never		Ever (1–2)		Many times (3–5)		Almost always (6 or more)		Always	
	*f*	*%*	*f*	*%*	*f*	*%*	*f*	*%*	*f*	*%*
Controls your schedule and/or decides what things you can do	251	77.7	44	13.6	13	4.0	8	2.5	7	2.2
Controls your money	297	92.0	17	5.3	4	1.2	3	0.9	2	0.6
Prevents you from having relationships with your family, friends, colleagues, etc.	245	75.9	38	11.8	24	7.4	9	2.8	7	2.2
Does not allow you to work or study	294	91.0	24	7.4	2	0.6	2	0.6	1	0.3
Controls your social networks and/or phone	228	70.6	54	16.7	24	7.4	7	2.2	10	3.1

Table 5 shows the results obtained in relation to the factor of Sexual Abuse, in which 80–91% of the participants stated that they had never received this type of mistreatment. The item “forcing you to have sexual relations against your will” was the most suffered abuse by the participants (14.2% had experienced this some of the time and 4.3% many times [3 to 5]). In this type of abuse, the option always was not selected.

**TABLE 5:** Sexual abuse.

Items	Never		Ever (1–2)		Many times (3–5)		Almost always (6 or more)		Always	
	*f*	*%*	*f*	*%*	*f*	*%*	*f*	*%*	*f*	*%*
Forces you to engage in sex that is degrading or humiliating to you	295	91.3	21	6.5	7	2.2				
Forces you to have sex against your will	259	80.2	46	14.2	14	4.3	4	1.2		

With regard to the analysis of the differences in the types of violence according to sex carried out using Student’s *t*-test for independent samples (Table 6), there were no statistically significant differences in relation to the five types of mistreatment analysed between women and men (ME, *T* = 0.773, *P* = 0.440; CSE, *T* = −1.635, *P* = 0.103; AS, *T* = 0.422, *P* = 0.674; MFP, *T* = −0.316, *P* = 0.752; DP, *T* = −0.456, *P* = 0.649). In this sense, the mean obtained for girls and boys in terms of the types of violence suffered in intimate relationships was not significantly different. Likewise, the averages obtained for each type of violence in relation to the sex variable indicate that women have higher arithmetic means than men in emotional and sexual abuse, personal devaluation, physical and psychological abuse, and social and economic control, in which a moderately greater difference can be observed (see Table 7).

**TABLE 6:** Student’s *t*-test for each type of violence in relation to sex.

		Levene’s test					
		Sig.	*t*	gl	Sig. (bilateral)	95% confidence interval of the difference	
						Lower	Upper
ME	Equal variances are assumed	0.169	0.773	321	0.440	−0.18914	0.43377
	Equal variances are not assumed		0.815	39.213	0.420	−0.18115	0.42578
CSE	Equal variances are assumed	0.095	−1.635	321	0.103	−0.39544	0.03651
	Equal variances are not assumed		−1.383	35.631	0.175	−0.44277	0.08383
AS	Equal variances are assumed	0.367	0.422	321	0.674	−0.12641	0.19535
	Equal variances are not assumed		0.549	45.483	0.586	−0.09191	0.16086
MFP	Equal variances are assumed	0.799	−0.316	321	0.752	−0.25517	0.18447
	Equal variances are not assumed		−0.368	41.649	0.715	−0.22912	0.15841
DP	Equal variances are assumed	0.343	−0.456	321	0.649	−0.25516	0.15921
	Equal variances are not assumed		−0.458	38.255	0.649	−0.25985	0.16390

**TABLE 7:** Comparison of means according to sex.

Gender		ME	CSE	AS	MFP	DP
Woman	Mean	1.5911	1.2955	1.1907	1.3271	1.2723
	*N*	291	291	291	291	291
	Standard deviation	0.85518	0.57519	0.44977	0.60928	0.56585
Male	Mean	1.4688	1.4750	1.1563	1.3625	1.3203
	*N*	32	32	32	32	32
	Standard deviation	0.80008	0.70893	0.32223	0.50402	0.56166
Total	Mean	1.5789	1.3133	1.1873	1.3307	1.2771
	*N*	323	323	323	323	323
	Standard deviation	0.84948	0.59096	0.43851	0.59908	0.56475

## 4. Discussion

Before presenting the conclusions and discussing the results, we would like to highlight the importance of the phenomenon studied and the bidirectional nature of violence. The aim of this research was to detect the presence of intimate partner violence in a sample of young students at the University of Granada, Spain, comparing sex with the possible forms of violence suffered.

When analysing the frequency of the types of violence in the participant sample, it was found that both men and women use violence in a bidirectional way. These results coincide with the results of research that indicates that regardless of sex, violence is exercised in young couple relationships, which begin with experiences that are difficult to perceive and that give rise to a process of escalating violence (Oliveira et al., 2014; Beserra et al., 2015; Martínez et al., 2016; Pérez-Ruíz et al., 2020; Quesada et al., 2020; Martínez-Gómez et al., 2021).

The descriptive analysis has identified a low-moderate trend in the responses, with the majority choosing the options “never” and “sometimes” and in which there were no statistically significant differences between girls and boys in terms of violence in intimate partner relationships (González-Gijón and Soriano, 2021). This coincides with the study by Hernando Gómez et al. (2012), which states that there are no gender differences in relation to physical and non-physical abuse in young university couples.

Emotional abuse and specific indifference to problems or needs have been identified as the most suffered types of abuse. As some authors have pointed out (Domenech del Rio and Sirvent García, 2016; Lara-Caba, 2019), young people tend to minimise this type of situation, producing harmful effects for those who suffer it, such as the deterioration of self-esteem and personal safety.

With regard to Physical and Psychological Abuse, the most experienced situation of violence occurs when, on certain occasions, their behaviour produces fear. This is followed by insults or threats, which coincide with psychological violence, with a high incidence in young people and which includes some types of violence related to Personal Devaluation, such as mistreatment based on a lack of consideration for religious and ideological beliefs and/or gender roles and stereotypes, which can be explained by the existing relationship between sexist beliefs and the increased risk of the use of psychological violence in both boys and girls (González-Gijón and Soriano, 2021; Guillén Verdesoto et al., 2021; Ruiz-Narezo et al., 2021).

Violence suffered through Social and Economic Control is identified with the young participants in the control of social networks and/or the telephone, data that coincides with the studies developed by Mera et al. (2021), in which 42% of the sample studied stated that they had suffered this type of violence from their partners on at least one occasion.

Finally, in Sexual Abuse, which appeared at a low prevalence, the situation most experienced by the participant sample is where sexual relations are forced against their wishes. Although studies show that men perpetrate more sexual violence (Schiff and Zeira, 2005; Fernández-Fuertes et al., 2006; Ortega et al., 2008; Munþoz Rivas et al., 2009; Rey-Anacona, 2013), in this study, there were no differences between the sexes.

## 5. Conclusion

From the results, it is concluded that there is a prevalence of violence based on emotional and psychological abuse in the context of the violence suffered, as it is culturally considered that, with this type of violence, you are not being abused (Pérez-Ruíz et al., 2020; Ruiz-Galacho and Martín-Solbes, 2020). In addition, partner abuse in young people is a phenomenon with a significant presence in interpersonal relationships, which occurs with bidirectional behaviour and affects gender symmetry and mutual violence.

Therefore, regardless of gender, young people are susceptible to intimate partner violence (Rodríguez Pérez, 2015). Hernández Hidalgo (2015), which urges us to recognise that the bidirectional phenomenon of intimate partner violence should not be understood as an attempt to deny, hide, or minimise the existence of violence against women.

For this, it is necessary to analyse the factors involved in this social phenomenon. Taking these results into account, it is vitally important to broaden the understanding of the violence that appears in the first relationships of young couples through the positioning of different methodological approaches to generate a new investigative approach to the phenomenon and be able to intervene through the design of socio-educational actions based on the prevention of mistreatment. The study has limitations in terms of the non-probabilistic sample used, which cannot be generalised to the rest of the study population.

## Data availability statement

The original contributions presented in the study are included in the article/supplementary material, further inquiries can be directed to the corresponding author.

## Ethics statement

The studies involving human participants were reviewed and approved by the University of Granada. The patients/participants provided their written informed consent to participate in this study.

## Author contributions

All authors participated in the preparation of this manuscript and agreed to be responsible for its content prior to publication.
